# Supplementary figures and images for: Triple-negative breast cancer influences a mixed M1/M2 macrophage phenotype associated with tumor aggressiveness
Source: PLoS One. 2022 Aug 12;17(8):e0273044. doi: 10.1371/journal.pone.0273044 (PMC9374254; doi:10.1371/journal.pone.0273044)

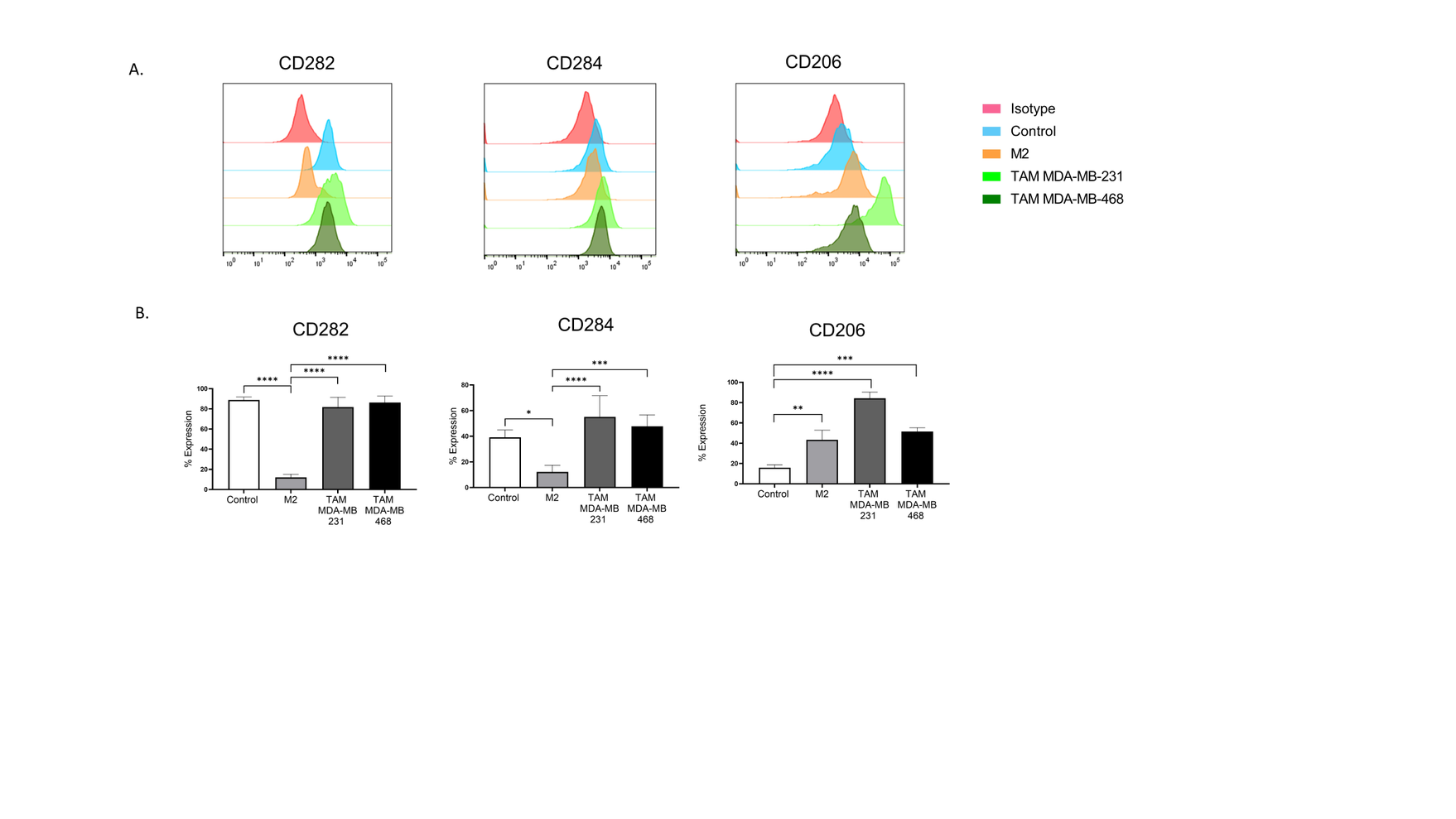

Supplement: S1 Fig — Monocyte are isolated from PBMC using CD14 magnetic bead. Primary CD14+ monocytes were incubated with RPMI media only (control), MDA-MB 231 conditioned media (TAMs MDA-MB-231), MDA-MB-468 conditioned media (TAMs MDA-MB-468), or IL-4 (20 ng/mL) and IL-13 (20 ng/mL) cytokines (M2) for 48 h. (A) Monocyte-derived macrophages expression level of M1 (CD282 and CD284) and M2 (CD206) surface markers were analyzed by flow cytometry as compared with an isotype control. (B) The bar graph represents the mean and SEM of M1 and M2 markers from three different experiments with significance level at *p < 0.05, *p < 0.05, **p < 0.01, and ***p < 0.001. (TIF) [file pone.0273044.s001.tif]
