# Supplementary material for: Triple-negative breast cancer influences a mixed M1/M2 macrophage phenotype associated with tumor aggressiveness
Source: PLoS One. 2022 Aug 12;17(8):e0273044. doi: 10.1371/journal.pone.0273044 (PMC9374254; doi:10.1371/journal.pone.0273044)
Supplement: S1 File — (DOCX) [file pone.0273044.s002.docx]

**Isolation of peripheral blood CD14^+^ monocytes**

PBMC were isolated by ficoll-paque gradient centrifugation (cytiva Cat no. 17544202) and CD14^+^ cells were magnetically labeled with CD14 microbeads (Miltenyi Biotec) for positive selection. Primary CD14^+^ monocytes were sorted by MACS separation technique according to the manufacturer’s instruction and seeded in a 24-well plate at a cell density of 2 x10 ^5^ cells/well. Primary CD14^+^ monocytes were incubated with RPMI media (supplemented with 10% FBS, 1% penicillin-streptomycin and 1X GlutaMAX) as control, MDA-MB 231 conditioned media (TAMs MDA-MB-231), MDA-MB-468 conditioned media (TAMs MDA-MB-468), or IL-4 (20 ng/mL) and IL-13 (20 ng/mL) cytokines (M2) for 48 h at 37°C in a humidified incubator with 5% carbon dioxide.

**Flow cytometry**

The surface staining of monocyte-derived macrophages was performed by staining with PE/Cy7 anti-CD80 (2D10), APC anti-CD86 (IT2.2), PE anti-CD163 (clone GHI/61), PE/Cy7 anti-CD206 (clone 15-2), FITC anti-CD68 (clone Y1/82A), FITC anti-CD282 (clone TL2.1), and PE anti-CD284 (clone HTA125) (Biolegend). Briefly, cells were harvested and washed with staining buffer and stained with antibodies for 15 minutes at 4°C. After staining, all cells were washed twice and analysis was carried out with flow cytometry (BD Accuri™).
