# Supplementary material for: Triple-negative breast cancer influences a mixed M1/M2 macrophage phenotype associated with tumor aggressiveness
Source: PLoS One. 2022 Aug 12;17(8):e0273044. doi: 10.1371/journal.pone.0273044 (PMC9374254; doi:10.1371/journal.pone.0273044)
Supplement: Attachment — Submitted filename: Review Comments.docx [file pone.0273044.s003.docx]

Review Comments:

Manuscript Title: "Triple-negative breast cancer influences a mixed M1/M2 macrophage phenotype associated with tumor aggressiveness."

Manuscript is written well and informative way. Data were clean and clear and followed the flow of writing.

I wonder that if authors also need to consider the protein level after stimulation of THP-1 cells with CM medium through western blotting which will tell more profound story of the manuscript.

I recommend that if authors could perform major proteins western blotting and showed the phenotypic condition in protein level that would be more readable.

After the minor revision of experiments this manuscript could be accepted in this journal
