# Supplementary material for: Triple-negative breast cancer influences a mixed M1/M2 macrophage phenotype associated with tumor aggressiveness
Source: PLoS One. 2022 Aug 12;17(8):e0273044. doi: 10.1371/journal.pone.0273044 (PMC9374254; doi:10.1371/journal.pone.0273044)
Supplement: Attachment — Submitted filename: Response to reviewers.doc [file pone.0273044.s004.doc]

Habib Boukerche, Ph.D

# Academic editor PLOS ONE

# May 12, 2022

Dear, Professor Boukerche

Manuscript number: PONE-D-21-39755

Title: Triple-negative breast cancer influences a mixed M1/M2 macrophage phenotype associated with tumor aggressiveness

Thank you very much again for carefully reviewing our manuscript and giving us very useful comments. We have made changes in the manuscript according to the editor/reviewer’ comments as the followings:

**Editor’s comments**

Comment #1: Data obtained with THP-1 cells should be validated with PBMC-derived primary macrophages which will be helpful to the improvement of the manuscript, in terms of clarifying the methodology and the goal of this manuscript.

Answer: We have performed an experiment on primary monocytes as editor’s suggestion. In the addition experiment, primary monocytes were treated with either conditioned media from two different TNBC cell lines or cytokines IL-4 and IL-13 for M2 induction, similarly to the THP-1 condition. We used flow cytometry to detect surface expression of M1 and M2 markers including TLR-2 (CD282) and TLR-4 (CD284) as M1 markers and CD206 for M2 marker. The results are described in the revised manuscript at page 10, line 235-240 and in a supplementary figure 1.

Comment #2: The authors should also show if such M1-associated genes induced by conditioned THP-1 cells is accompanied by expression of classical M1 surface markers. The authors also need to address  the protein level after stimulation of THP-1 cells with CM medium through western blot.

Answer: We agree that the incorporation of protein analysis would make the finding more profound. However, due to our laboratory limitation, we could not perform such experiments as recommended. Therefore, we have mentioned this limitation in the revised manuscript on page 19, line 467-474. Also, the M1/M2 mixed phenotype on TNBC-induced TAMs in our study were further verified by surface expression of M1 and M2 markers on primary monocytes that were conditioned with TNBC cultured media. Moreover, analysis of two independent patient database follows our in vitro finding where M1 associated genes are upregulated in TNBCs particularly the key marker CXCL-10 which is negatively correlated with disease-free survival.

**Reviewer 1’s comments**

Comment#1: As a general comment, I find it difficult to accept that a tumor cell line (THP-1 cells) conditioned with TNBC media could be compared to TAMs. At least the results obtained with THP-1 cells should be validated with PBMC-derived primary macrophages.

Answer: As mentioned earlier, we have performed an experiment on primary monocytes as recommended. The results are described in the revised manuscript at page 10, line 235-240 and in a supplementary figure 1.

Comment#2: First of all, conditioned THP-1 cells express M1-associated genes without reporting the indicated markers from a classically-stimulated positive control. Except for CXCL10 and TGF1B, ThP-1 conditioning does not induce a significant increase for any of the markers indicated in Figure 2, when compared to the corresponding control. So it is not clear if these conditioned cells have reached their polarization status or not. Then Figure 3 shows a relevant increase in the IL-6 , IL-10 and TNFa release by TNBC-conditioned cells, so these data should be included in Figure 2.

Answer: In addition to M1 and M2 associated gene expression, the characterization of TNBC-conditioned cells was also determined with cellular morphology and secreted cytokines. In the revised manuscript, we have combined the figure 2 and 3 as suggested.

Comment3#: It is also not clear in Figure 2 the media of which cancer cell type was used to condition ThP-1 cells.

Answer: The conditioned media from MDA-MB-231 cells were used to treat PMA-stimulated THP-1 for M1- and M2- associated gene expression and morphological observation. This was also clarified in the figure legend of figure 2 in the revised manuscript.

Comment 4# Additionally, ThP-1 alternative polarization (labelled as M2, obtained with stimulation with IL-4+IL13 I believe) does not induce a significant increase of the typical associated markers (Figure 2), which is also quite strange. Since IL-4 and IL-13 produce different effects, the indicated markers should be evaluated in cells stimulated with IL-4 alone and IL-13 alone.

Answer: We used the combination of IL-4 and IL-13 to polarize M2 macrophage based on the earlier report by Genin et al. ([*BMC Cancer*](https://bmccancer.biomedcentral.com/) volume 15, Article number: 577 (2015)) in which we consistently observed the upregulation of CCL22 and CCL18. The extent of increase, however, is different between our study and theirs. The different stimulation time, passages of cell lines and culture conditions could attribute to this discrepancy between studies.

Comment #5: Additionally, data regarding the expression of M1 and M2-related metabolic enzymes, such as iNOS, PKM2 or ARG1 should also be included.

Answer: Although, in-dept characterization of classical M1 and M2 macrophage would be ideal for the current manuscript, the primary objective of the current study is to demonstrate the complexity and heterogeneity of TAMs induced by TNBC which were evidenced by common M1 and M2 gene expression, morphology, and secreted mediators as well as the confirmation of surface markers in primary monocytes.

Comment #6: To evaluate the functional features of these conditioned ThP-1 cells, some migration experiments were performed finding that TAMs and M2-polarized macrophages increased the motility of TBNC cells. In the same way, the authors should study the capacity of these conditioned cells to increase the invasive properties of TBNC cells and analyze the expression levels of classical migration/invasion markers such as N-cadherin, E-cadherin or vimentin.

Answer: We have used migration and proliferation assay as a functional study to confirm the negative effects of our TNBC-induced TAMs model on breast cancer. Previous studies have also shown the effects of TAMs on the increased expression of migration/invasion markers on various types of cancer (Gan et al., 2016, Wei et al., 2019, Yao et al., 2018)

Comment #7: Also, since these cells exhibit a significant increase in the CXCL10 expression levels, the ability of these cells to induce CD8+ T cell recruitment should be reported.

Answer: The effect of TAMs to immune cells is out of scope for this manuscript.

Comment #8: Page 3, line 40 -42, In this paragraph this sentence is written: “Accumulated evidence suggests that TAMs are a heterogeneous and plastic population, in which polarized TAMs can be identified as M1- and M2-like macrophages”. Please reference.

Answer: Reference were done. Page 3, line 48

Comment #9: Line 219 Please write the full name for the acronym BCA.
Answer: Correction has been done. Page 11, line 264

Comment #10: Figure legends: please specify the cell types and the treatment each result comes from.
Answer: The figure legend has been revised.

Comment #11: Methods: please describe the transwell assay with more detail.

Answer: More description of the Transwell assay has been provided. Page 6 Line 118-127

**Reviewer 2’s comments**

Comment#1: Manuscript is written well and informative way. Data were clean and clear and followed the flow of writing. I wonder that if authors also need to consider the protein level after stimulation of THP-1 cells with CM medium through western blotting which will tell more profound story of the manuscript. I recommend that if authors could perform major proteins western blotting and showed the phenotypic condition in protein level that would be more readable.

Answer: We agree that the incorporation of protein analysis would make the finding more profound. However, due to our laboratory limitation, we could not perform such experiments as recommended. Therefore, we have mentioned this limitation in the revised manuscript on page 19, line 467-474. The M1/M2 mixed phenotype on TNBC-induced TAMs in our study were verified by gene expression, morphology, and cytokines profile of THP-1 derived macrophage as well as surface expression of M1 and M2 markers on primary monocytes. Moreover, analysis of two independent patient database follows our in vitro finding where M1 associated genes are upregulated in TNBCs particularly the key marker CXCL-10 which is negatively correlated with disease-free survival.

Sincerely,

Supannikar Tawinwung

Corresponding Author
